# Proteomic analysis of pancreatic endocrine cells by mechanistic single-cell isolation identifies membrane pathways

**DOI:** 10.1038/s12276-026-01826-9

**Published:** 2026-09-03

**Authors:** Lihi Grinberg, Worood Sirhan, Marina Kurtz, Michael Assa, Assaf Malka, Shira Perez, Corine Katina, Yishai Levin, Ron Piran

**Affiliations:** 1 The Regenerative Medicine and Diabetes Laboratory, The Azrieli Faculty of Medicine, Bar-Ilan University, Safed, Israel; 2 The Genomic Center, The Azrieli Faculty of Medicine, Bar-Ilan University, Safed, Israel; 3 The Microscopy Center, The Azrieli Faculty of Medicine, Bar-Ilan University, Safed, Israel; 4 The Nancy and Stephen Grand Israel National Center for Personalized Medicine, The Weizmann Institute of Science, Rehovot, Israel

**Keywords:** Proteomic analysis, RNA sequencing, Diabetes, Proteomics

## Abstract

To better understand diabetes and normoglycemia, pancreatic islet biology requires a precise molecular understanding of islet cell types at both the transcriptomic and proteomic levels. While transcriptomic analyses are well established, comprehensive proteomic characterization has been lacking, limiting our knowledge of islet molecular complexity. Here we introduce a nonenzymatic, mechanistic single-cell isolation technology using laser microdissection (LMD7), facilitating proteomic and transcriptomic analysis of physically isolated α-, β- and δ-cells from fresh-frozen, unfixed pancreatic tissue. This mechanistic approach avoids enzymatic digestion and chemical fixation, preserving the cells’ native molecular state before processing. Given the limited existing proteomic data, we supplemented our findings with transcriptomic analysis generated using the same method and compared our results with data from enzymatically isolated cells, obtained by fluorescence-activated cell sorting and compiled by others. Our analysis revealed that enzymatic digestion alters gene expression patterns, particularly those of membrane-associated proteins, underscoring the impact of isolation techniques on biological outcomes. We identified cell-type-specific proteins typically underrepresented in pancreatic single-cell transcriptomic datasets. β-cells exhibited enrichment in vesicle trafficking proteins, α-cells displayed distinct calcium-dependent action potential machinery and δ-cells showed elevated expression of focal adhesion-related proteins. In addition, we report an inverse molecular relationship between β- and δ-cells, potentially driven by transcriptional regulators such as Mlxipl. By establishing robust molecular profiles directly from intact pancreatic tissue, this work provides a reference point for future pathological comparisons, offering a framework to investigate how diabetes and other endocrine disorders reshape islet cell biology.

## New method reveals complex proteomic profiles of islet cells

This study explores the complex cellular composition of pancreatic islets, revealing distinct functional characteristics of α-, β- and δ-cells that are relevant to diabetes research. Researchers developed a novel method with a laser microdissection-based workflow for single-cell proteomic and transcriptomic analysis, overcoming limitations of traditional enzymatic cell separation, which can degrade membrane proteins and alter gene expression. By rapidly freezing tissue and using laser microdissection, they preserved the native molecular state of cells, allowing for accurate profiling. The study identified unique protein and gene expression patterns in each cell type, showing that α-cells are enriched in calcium signaling pathways, β-cells in vesicle trafficking and δ-cells in focal adhesion pathways. These findings suggest that pancreatic endocrine cells have specialized roles beyond hormone secretion, acting as sensors and regulators within the islet microenvironment.

This summary was initially drafted using artificial intelligence, then revised and fact-checked by the author.

## Introduction

Diabetes mellitus is a group of diseases characterized by insufficient insulin signaling. This insufficiency can arise from two main causes: (1) insufficient insulin production, as observed in type 1 diabetes (T1D), resulting from an autoimmune attack on the insulin-producing β-cells^1,2^; and (2) insulin resistance, where insulin signaling is impaired^3^. Initially, insulin resistance leads to elevated insulin secretion, as observed in the early stages of type 2 diabetes (T2D), which eventually results in chronic insulin production and β-cell exhaustion^4^. Consequently, both T1D and T2D culminate in pancreatic β-cell deficiency, albeit through different mechanisms.

The pancreatic β-cells, along with their neighboring α-, δ-, ε- and pancreatic polypeptide (pp) cells, as well as other structural cells, compose the islet of Langerhans^5,6^. These complex and heterogeneous structures are responsible for the endocrine function of the pancreas, that is, the regulation of bodily glycemic levels^7^. The unique architectural structure of these islets varies between organisms. In humans, islet cell types are highly interconnected, where β-cells are typically associated with at least one non-β-cell. By contrast, mouse islet architecture follows a core-mantle organization, with a β-cell-rich core surrounded by a mantle of the other endocrine cell types^8^. Moreover, accumulating evidence indicates that individual islet cell types may function differently depending on their specific location within the islet structure^9,10^, highlighting the importance of islet architecture and intra-islet communication between the islet cell types in regulating their function^11^.

**Fig. 1:**
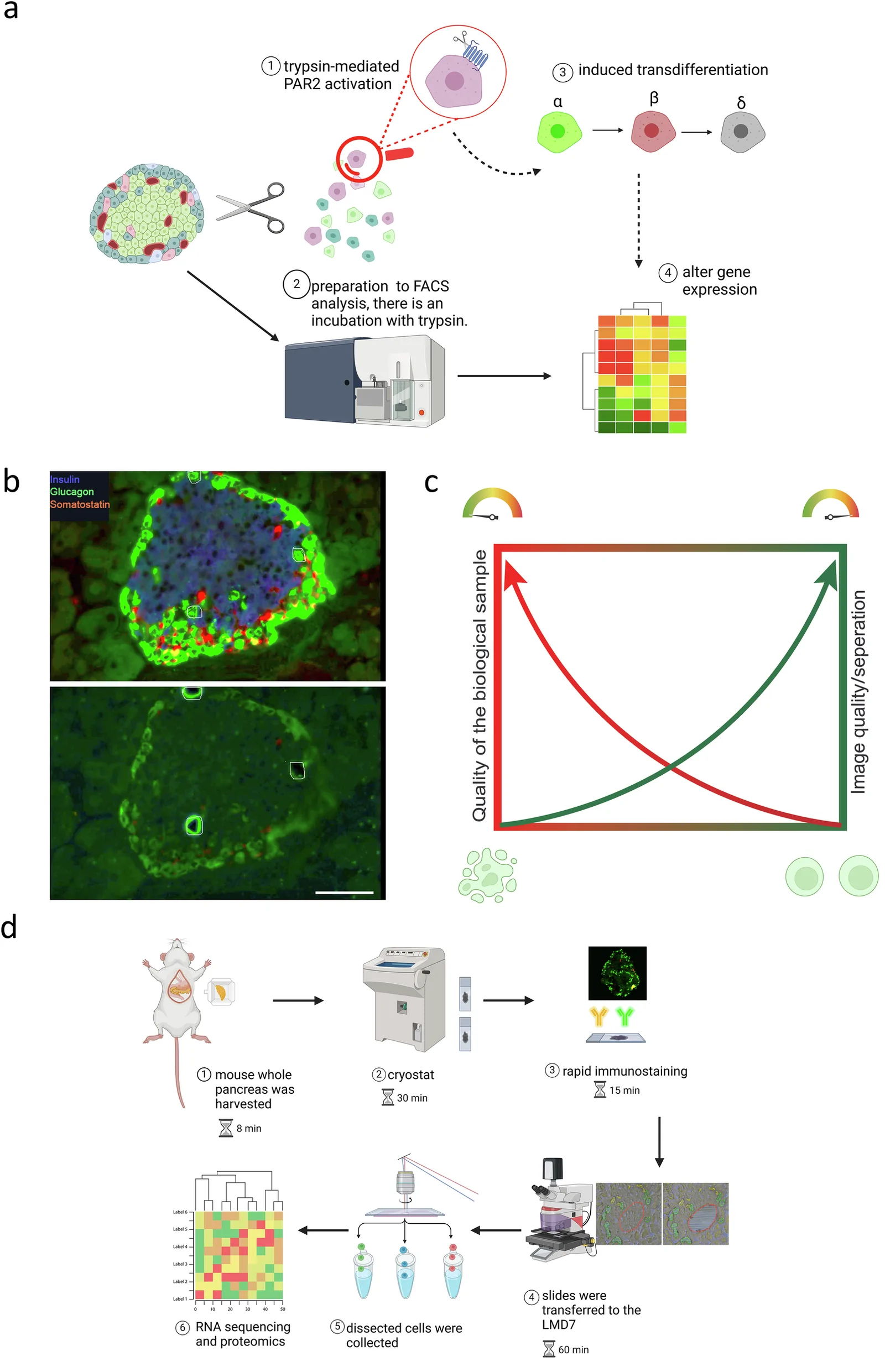
FACS and flow cytometry methodology versus mechanistic separation by LMD. **a**, A schematic depiction of the experimental processes required to obtain transcriptomic data using FACS. This illustration highlights that trypsin and trypsin-like enzymatic separation of different cells may lead to changes in gene expression, such as Par2 activation. **b**, The initial immunostaining of α-, β- and δ-cells according to glucagon (green), insulin (blue) and somatostatin (red) staining, respectively, in **a**. This clear staining allowed perfect cell separation, as demonstrated by the specific exclusion of individual α-cells in the lower panel (images acquired by the LMD7). **c**, The contention between achieving a perfect image and acquiring biological data. While perfect islet immunological staining was achieved, no meaningful biological data were extracted from these samples. The graph in **c** showcases the contention between obtaining a perfect image with no biological information and an unprocessed sample with full biological data that cannot be separated into cell types. One needs to find the best compromise between these two needs to obtain optimal information from individual cell types. **d**, A schematic depiction of our modified sample processing method to achieve maximal biological data. Mouse pancreata were processed to the block stage within 8 min. Slides were prepared within 30 min, and rapid immunostaining was completed within 15 min. Subsequent processing in LMD was completed within an hour, allowing proteomic and transcriptomic α-, β- and δ-cell characterization.

Previous studies have extensively characterized islet transcriptomics^12–14^, some using laser capture microdissection techniques^15,16^. Thus, initial islet transcriptome characterizations represented an average of all cell populations. The advent of single-cell RNA sequencing (scRNA-seq)^17,18^ has enabled the profiling of mRNA content in individual cells from heterogeneous tissues. This technique allows for the characterization of diverse cell types within the pancreatic islets of Langerhans. Following these early studies, more precise cell-type oriented transcriptomic analyses were conducted, mainly by fluorescence-activated cell sorting (FACS). Nonetheless, to create a single-cell suspension, cells must be dispersed into single cells in solution (Fig. 1a).

Although single-cell sequencing has become increasingly standardized^19^, the complementary assessment of single-cell proteomics is lagging behind, with limited progress in islet biology. Recent works on islet proteomics are still in the whole-islet stage, and only now are averaged islet proteomes being deciphered^20,21^, with a recent study finally reporting such analyses in human islets^22^. Proteomic resolution at the level of individual islet cell types is only now emerging^23^, enabling preliminary comparisons of molecular signatures with pathological alterations in diabetes.

One reason for the slow progress in islet proteomics is the method used for islet cell separation. This process is traditionally performed using trypsin to dissociate the cells. However, trypsin also digests proteins, specifically stripping membranal proteins from the cell surface and, as a result, hides them from the analysis. In addition, trypsin is a constitutive Par2 activator that affects cellular expression^24^. Previously, we identified the protease-activated receptor 2 (Par2) as a prominent mediator of a β-cell transdifferentiation pathway from neighboring α-cells^25,26^, positioning it as a global regulator of inflammation and regeneration^24,27^. In the pancreas, Par2 activation, predominantly by trypsin, induces α-cells to express β-cell markers within minutes^25^. In our transdifferentiation model, however, newly formed β-cells were unstable and continued to differentiate into somatostatin-expressing δ-cells^28^.

To halt the transdifferentiation process of α- to β- to δ-cells at the β-cell stage, we aimed to target δ-cell-specific biochemical pathways. However, little is known about δ-cells, as they are not linked to any single pathology and have historically been overlooked in diabetes research. This undercharacterization itself represents a limitation in pathology, as δ-cells may play a stabilizing or destabilizing role in islet architecture and in β-cell failure. Therefore, our goal was to characterize pancreatic δ-cells and map their differences from adjacent α- and β-cells.

In the present study, we introduce a technology using the laser microdissection (LMD) apparatus for proteomic and transcriptomic single-cell analysis of islet cell types and compare these findings to previously reported transcriptomic results obtained by others using different methodologies. Moreover, it is known that gene expression in cells may change when they are removed from their natural tissue environment^25,29,30^. Thus, cryofixing the cells using the fresh-frozen technique before separation and isolation is beneficial for obtaining a tissue snapshot. In addition, LMD technology enables proteomic characterization of individual cell types, a profiling method that, to our knowledge, has never been performed in a heterogeneous environment such as pancreatic islets. This advantage can be extended to other dense biological tissues composed of various cell types and provides a needed molecular framework for distinguishing healthy from pathological states. The primary objective of the present study was to establish and validate a mechanistic isolation and multi-omic profiling framework under controlled physiological conditions, to define reference molecular profiles for pancreatic endocrine cell types, and to use this framework to uncover biological features that may be obscured by conventional enzymatic workflows. Application of this framework enabled the discovery of membrane-associated pathways and additional molecular signatures that characterize endocrine cell identity and function.

## Materials and methods

### Animal handling

All animal experiments were conducted in accordance with institutional and national guidelines for the care and use of laboratory animals and were approved by the Institutional Animal Care and Use Committee (IACUC) of Bar-Ilan University. Inbred CFW and C57BL/6 mice, aged 3 months, were housed under standard laboratory conditions with a 12-h light–dark cycle, controlled temperature and humidity and free access to food and water. The animals were acclimatized for at least 1 week before the start of the experiments. All efforts were made to minimize suffering and reduce the number of animals used in the study.

### Tissue blocking and sectioning

Mice were anesthetized for 2 min with isoflurane (100% UPS; Piramal Critical Care), after which the pancreas and spleen were harvested within 6 min at room temperature, with a focus on the area connected to the stomach without excess fat tissue. Tissue samples were embedded in optimal cutting temperature (OCT) compound (Scigen) for transcriptomic analysis or 1 M sucrose (cat. no. S0389 Sigma-Aldrich) in double-distilled water for proteomic analysis, both at room temperature, and were rapidly fresh frozen in liquid nitrogen. The sucrose-based embedding matrix was developed as an alternative to OCT for proteomic applications, providing adequate tissue support during cryosectioning while maintaining compatibility with downstream mass spectrometry (MS) analyses. The embedded tissues were then stored at −80 °C for 30–120 min until sectioning on the same day. Before cryostat sectioning, the Leica CM3050S cryostat (Leica Biosystems) was cleaned with 70% ethanol to remove any RNA and protein contaminants, and the slides were ultraviolet (UV)-sterilized in a biological safety cabinet (LABCONCO, Logic Vue Configurator). The cryostat was set to −17 °C, and sectioning was performed to obtain 5-µm-thick sections. All cryosections were completed within 30 min. Sections were collected onto specially designed slides for LMD: positron-emission tomography (PET)-membrane 1.4-µm slides for transcriptomic analysis (Leica Microsystems GmbH) and Director slides for proteomic analysis (cat. no. 50001-024, Leica Microsystems GmbH). The slides were then transferred to a biological safety cabinet, fixed with 1,6-hexanediol (cat. no. H11807; Sigma-Aldrich) in methanol (9:1 vol/vol) (Macron Fine Chemicals), placed on ice for 2 min and air-dried for 1 min using a hair dryer set to ventilation mode at room temperature. The slides were placed on a plastic slide box located inside the cryo-chamber set to −17 °C, and additional sections were cut. After that, the slides were transferred to −20 °C until all cryostat sectioning was finished. For every block change, the blade of the cryostat was changed to avoid contamination. The sections were stored at −80 °C until further use. All sections were handled carefully to preserve tissue morphology and biological quality.

### Standard immunostaining

For regular immunostaining, pancreatic tissue from mice was fixed in 4% paraformaldehyde (PFA) in PBS (Santa Cruz Biotechnology) overnight at 4 °C, followed by thorough washing with PBS at room temperature, (Biological Industries) and subsequent overnight incubation in 30% sucrose (cat. no. S0389; Sigma-Aldrich) at 4 °C. The tissue was then embedded in OCT compound (Scigen) and frozen in a mixture of dry ice and 2-methylbutane (cat. no. 106056; Sigma-Aldrich). Frozen tissue blocks were sectioned at a thickness of 5 µm using a Leica CM3050S cryostat set to −20 °C (Leica Biosystems).

Before use, all tools, brushes and gloves were sterilized with a UV light source, and the cryostat was cleaned with 70% ethanol. The cryosections were air-dried and washed three times with PBS, followed by incubation in 0.3% Triton X-100 (MP Biomedicals) in PBS for 15 min at room temperature. After additional washes with PBS for 15 min, the sections were blocked with 5% normal donkey serum (Jackson ImmunoResearch) for 45 min at room temperature. The cryosections were then incubated with primary antibodies specific for insulin (1/400, cat. no. 17666-20B, guinea pig; US Biologicals) glucagon (1/400), rabbit, (cat. no. NBP2-38330; Novus Biologicals) and somatostatin (1/400, cat. no. NB100-64650; Novus Biologicals) overnight at 4 °C followed by secondary antibodies conjugated to Alexa Fluor 488 (cat. no. 016-540-084), AMCA (cat. no. 016-150-084) and rhodamine red-X (cat. no. 106-240-085) at room temperature, which were purchased from Jackson ImmunoResearch.

### Rapid immunostaining

For rapid immunostaining, solutions were freshly prepared and supplemented with RNase inhibitor (Promega Corporation) to prevent RNA degradation, in a biosafety cabinet (LABCONCO) without airflow. The primary antibodies used were antiglucagon (GLU) (cat. no. NBP2-21803AF594; Novus Biologicals), conjugated to Alexa Fluor 594 and somatostatin (SST) antibody (cat. no. sc-47706; Santa Cruz Biotechnology) conjugated to Alexa Fluor 488. High concentrations of primary antibodies (2:25 dilution for GLU, 1:4 dilution for SST), along with 10% donkey serum (Jackson ImmunoResearch) and RNase inhibitor (400 U/ml; RNasin Ribonuclease Inhibitor, Promega), were premixed for 15 min at room temperature. Before staining, each tissue section was dried with clean absorbent wipes. A PAP-pen (cat. no. ab2601; Abcam) was used to draw a barrier around each tissue section on the slides, taking care to avoid pressing too hard to prevent membrane damage. This step was conducted at room temperature. The sections were rapidly washed twice for 10 s each with PBS (cat. no. P3813; Millipore Sigma) at room temperature, dehydrated, air-dried and blocked with 10% donkey serum (Jackson ImmunoResearch) in PBS for 5 min. Staining was performed for 15 min at room temperature to minimize RNA and protein degradation. This abbreviated staining protocol was specifically developed to preserve molecular integrity while maintaining sufficient fluorescent signal for reliable identification of endocrine cell types during LMD.

Each slide was processed individually for rapid immunostaining, followed by immediate LMD at room temperature under low-humidity conditions as described below. After completing the LMD procedure on one slide, the next slide was stained and processed similarly. This approach ensured that only one slide was handled at a time to maintain sample integrity and prevent degradation. The sequential processing strategy minimized ex vivo exposure time and reduced the potential for isolation-induced molecular alterations before cell collection. Immunofluorescent staining for proteomic analysis was conducted similarly but without the RNAse inhibitor.

### LMD following immunostaining

Once stained, the unmounted dry slide was transferred to the Leica LMD7 (Leica Microsystems GmbH) and placed according to the manufacturer’s protocol. Before and after images of each section were obtained using the proprietary Leica LMD7 software at 20× magnification. Three LMD7 0.5-µm Eppendorf tubes (Leica Microsystems GmbH) were placed under the target area so that each cell type could be collected in its own vial. Pulsing a laser close to the sample led to melting of the thermoplastic film, causing the target cells of interest to separate from the membrane. The dissected cells were collected directly into the cap of a 0.5-µm Eppendorf tube.

For proteomic analysis, a cutting method named ‘cut and scan’ was used, as described in Supplementary Movie 3, which specifies the process for Director slides. During this process, UV heating was applied to the slide. The UV heating did not damage the slide but facilitated the flipping of the dissected cells down into the open Eppendorf cap. For proteomic extraction, the Eppendorf cap was covered completely with 2,2,2-trifluoroethanol (cat. no. 808259; Sigma-Aldrich) and placed at −80 °C for further analysis. A total of 155 pancreatic islets were dissected, from which ~4100 α-cells, ~20,000 β-cells and ~600 δ-cells were collected. The different cell types were distributed into 36 individual Eppendorf cups, with each tube containing an average of ~700 islet cells of a single cell type.

For RNA extraction, 50 µl extraction buffer from the PicoPure RNA Isolation Kit (cat. no. KIT0204; Thermo Fisher Scientific) mixed with 2.5 µl SUPERase•In (cat. no. AM2694; Invitrogen, Thermo Fisher Scientific) was placed in an Eppendorf cap. Each LMD7 session did not exceed 40 min in a low-humidity area. A total of 104 pancreatic islets were dissected, from which ~4800 α-cells, ~8400 β-cells and ~600 δ-cells were collected. The different cell types were distributed into 36 individual Eppendorf cups, with each tube containing an average of ~400 islet cells of a single cell type.

### MS-based proteomics

Extracts were dried and then co-reconstituted in 4 ml 50 mM ammonium bicarbonate and kept on ice. A total of 7.5 ng trypsin was added (without reduction and without alkylation) for 4.5 h at 37 °C. Digestion was stopped by the addition of 0.1% formic acid and subjected to nano liquid chromatography (LC)–tandem MS (MS/MS). Peptides were analyzed using the nanoElute2, kept at 10 °C. The mobile phase comprised 0.1% formic acid in water (A) and 0.1% acetonitrile (B). Peptides were separated using a Symmetry trap (0.180 × 20 mm, Waters) and a 0.075 × 250-mm analytical column (Aurora, Ion Optiks), with the column at 50 °C and using the following gradient: 2% B to 30% B in 60 min, 30–95% B in 0.5 min, maintained for 5 min. Raw data were acquired with a timsTOF Pro (Bruker Daltonics) in data-independent acquisition parallel accumulation–serial fragmentation (DIA-PASEF) mode. The mass range was 306.8–1,195.8 Th; mobility range, 0.63–1.42 1/K_0_; and collision energy, 20 and 59, respectively.

Raw data were processed using Spectronaut v17.6 with the default parameters. Data were searched against the mouse sequences from UniprotKB/Swissprot version 01_2024.

### RNA isolation

The RNA PicoPure RNA Isolation Kit (Arcturus, Thermo Fisher Scientific) was used with the following modifications: a spin column-based method was used with a DNase step (RNase-Free DNase Set, cat. no. 79254; QIAGEN) with small adjustments. Before incubation, the samples were cocentrifuged at 3000*g* for 30 s. In the final step, 12 µl elution buffer mixed with 0.6 µl SUPERase•In (Invitrogen, Thermo Fisher Scientific) was added directly onto the membrane column and incubated for 7 min.

### Library preparation

RNA library preparation and sequencing were performed at the Genome Technology center at the Azrieli Faculty of Medicine Bar-Ilan University, Israel. Libraries for Illumina sequencing were performed using the SMART-Seq Stranded Kit (Takara Bio USA) according to manufacturer’s instructions. Quantification of the library was performed using dsDNAHS Assay Kit and Qubit 2.0 (Molecular Probes, Life Technologies) and qualification was done using the Agilent D1000 Tapestation Kit and Tapestation 4200 (Agilent Technologies). A total of 300 nM of each library was pooled together and diluted to 4 nM according to NextSeq manufacturer’s instructions. Then, 1.5 pM of the pool was loaded onto the Flow Cell with 1% PhiX library control. Libraries were sequenced on an Illumina NextSeq 500 instrument using 75-cycle single-read sequencing.

### Sequencing data processing and differential gene expression for transcriptomic analysis

Single-cell RNA-seq data were processed and converted to gene read counts using Partek Flow (Partek). Fastq files were aligned to the mm10 mouse reference genome (GRCm38/mm10) using the Cell Ranger pipeline (v3.0.2, 10x Genomics) with default parameters. The gene read counts were normalized across samples using the Total Count method. Genes with an average coverage of ≥1 read per million were defined as expressed and selected for differential gene expression analysis using the Reads Per Kilobase of exon per Million mapped reads method. All the statistical analyses were performed in RStudio (R Foundation for Statistical Computing). The ggplot2 package was used for data visualization, and the dplyr package was used for data manipulation. The confidence in the network meta-analysis effect estimates for all outcomes and treatment comparisons was evaluated using these R packages.

### Data comparison

The comparison of the data between the ‘proteomic’ and ‘transcriptomic’ datasets was conducted using a structured approach to evaluate the similarities and differences. Both datasets were formatted into Excel files with consistent structures, and each gene’s expression value was recorded. Genes in the proteomic dataset were ranked from highest to lowest on the basis of their expression values, with the highest expression assigned a rank of first place and the lowest expression receiving the last place ranking number. The same ranking process was applied to the transcriptomic dataset. The expression values were then normalized by dividing each gene’s expression place on the list by the total number of participants on that list, resulting in a relative expression level for each gene, with first place receiving the lowest value and the last place receiving the value of 1 (Supplementary Excel Sheet 3).

A threshold of |0.15| was applied to determine similarity. Genes with differences in normalized expression levels within this range were considered similar and assigned a value of 1. Differences beyond this threshold were marked as different and assigned a value of −1. Genes present in the transcriptomic data but not in the proteomic data were assigned a value of 0.5, and genes present in the proteomic data but not in the transcriptomic data were assigned a value of −0.5. The analysis was performed using Excel functions to compare gene expression values between the two datasets, with the numeric values recorded in a new column for each gene and protein (Supplementary Excel Sheet 3). The processed data were then imported into RStudio (R Foundation for Statistical Computing) for further statistical analysis.

A similar analysis was conducted for our results in transcriptomic and proteomic datasets versus the van Gurp et al. datasets^19^, with adjustments for different thresholds and expression types. In the transcriptomic analysis, thresholds of |0.3| and |0.45| were also applied. For the van Gurp et al. datasets, genes were ranked on the basis of their total of transcript (TOT) values, where the highest TOT value was 21. Each gene’s TOT was divided by 21 to obtain a relative expression level. This normalized expression level was then used to determine similarities and differences with the same criteria and thresholds as described previously (Supplementary Excel Sheet 4).

The proteomic data generated in this study were compared with results reported by Thielert et al.^23^, which encompass more than 6000 proteins per cell type. As not all protein measurements in that dataset were consistent across replicates, we restricted the comparison to proteins displaying a standard deviation ≤0.25 (Supplementary Excel Sheet 7). For this analysis, we applied the same 1, 0.5, −0.5 and −1 scoring indexes described above, using the stringent |0.15| threshold.

## Results

### The quality dilemma: image quality versus biological quality

To isolate α, β and δ-cells using LMD, proper histology is required. C57BL/6 PFA-fixed pancreata were embedded in OCT, sectioned by a cryostat on PET-membrane 1.4-µm slides (Leica) and immunohistochemically stained overnight with primary antibodies against glucagon, insulin and SST to identify α-, β- and δ-cells, respectively. This staining was followed by incubation with fluorescently labeled secondary antibodies. A clear resolution was achieved between the three endocrine cell types, which were successfully separated to create homogeneous single-cell populations (Fig. 1b, Supplementary Fig. 1 and Supplementary Movies 1 and 2). However, the quality of the biological data was poor. The extracted RNA was degraded, and no proteins could be detected. We reasoned that our samples were severely damaged owing to prolonged incubation and chemical fixation times, leading us to optimize the process (Fig. 1c).

Several aspects contribute to the ‘perfect image’. For regular immunofluorescence staining, PFA incubation is imperative. After PFA, the tissue is transferred into a sucrose solution to equilibrate the conditions inside and outside the sampled tissue and mimic the osmotic conditions of the OCT block. After cryosectioning, the slides are carefully rinsed of OCT traces and a slow process of exposures to different antibodies is initiated. This process typically takes approximately 1.5 days.

These challenges are even more pronounced in the pancreas, as ~95% of the tissue is composed of acinar cells containing digestive enzymes^31^. Following overnight PFA incubation, all enzymes are inactivated and all proteins are fixed in their positions. Skipping this stage in the pancreas is unthinkable, as acinar digestive enzymes, particularly ribonucleases and proteases, can lead to the loss of biological data. While PFA incubation enhances tissue integrity and imaging clarity, its cross-linking properties hinder downstream sequencing of both RNA and proteins. As a result, we opted to fix our samples by fresh freezing instead (‘Materials and methods’ section).

Owing to the lack of biological results, we realized that our process must be much faster and PFA free. Pancreata were harvested within 8 min and immediately transferred into blocks cooled by liquid nitrogen fumes. Slides were prepared and followed by rapid immunostaining. The LMD processing time was also minimized (Fig. 1d). These rapid procedures resulted in meaningful data.

### Mouse comparison

**Fig. 2:**
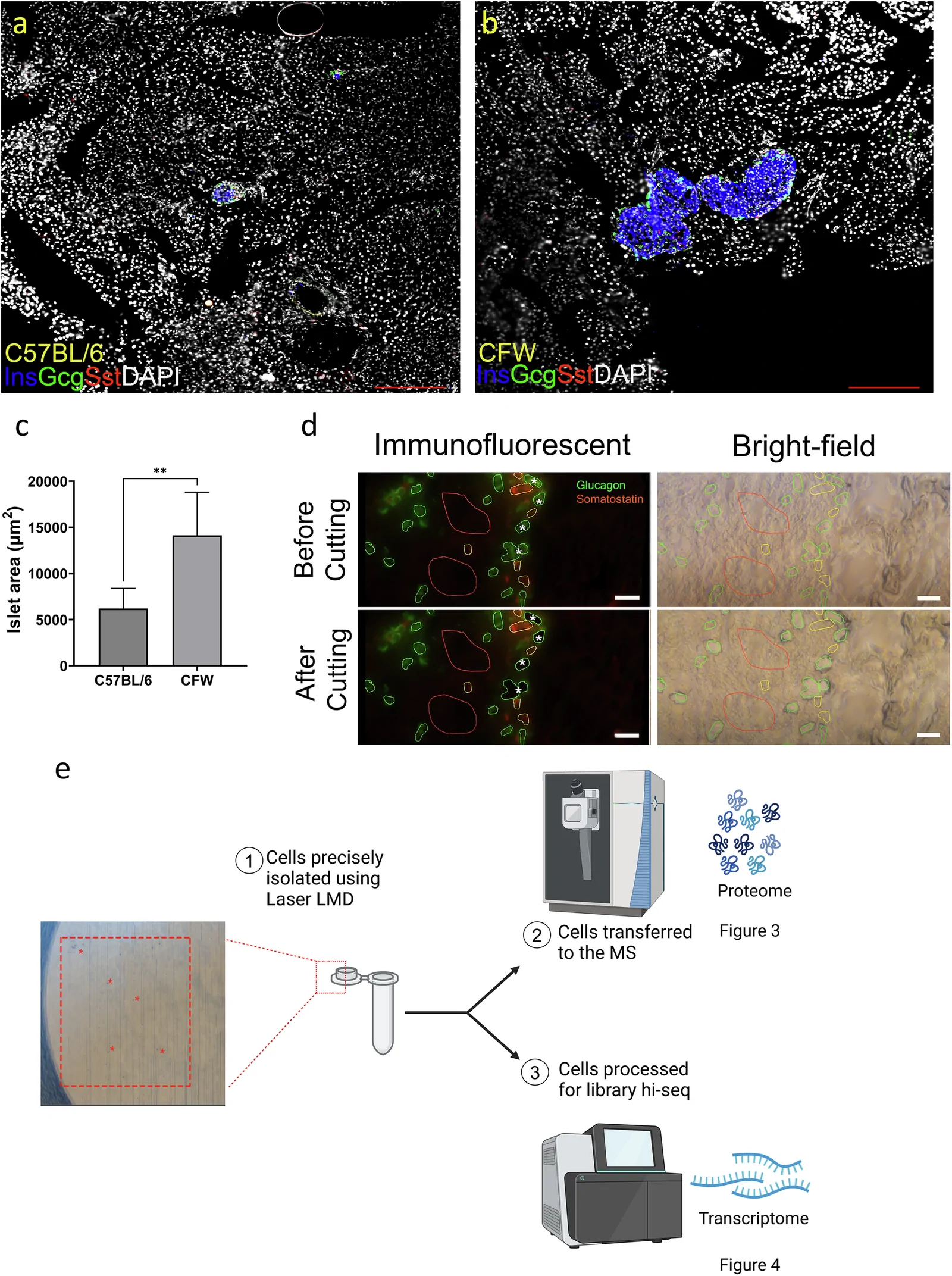
Choosing the right strain for the LMD mechanistic separation method. **a**,**b**, Immunofluorescence images of pancreata taken from the C57BL/6 (**a**) and from the CFW (**b**) mouse strains. Glucagon in green, insulin in blue, SST in red and 4,6-diamidino-2-phenylindole (DAPI) in white. **c**, Islet area quantification between the C57BL/6 and CFW mouse strains (*n* = 7 mice from each strain). The islet area was significantly different between the two strains (*P* < 0.01, by Student’s *t*-test). **d**, A sample-mounted PET slide before (top) and after (bottom) specific α-cell excision. Rapid immunofluorescence images showing α-cells in green (stained for glucagon) and δ-cells in orange (stained for SST) on the left and bright-field images on the right. **e**, A schematic illustration of the cells taken for analysis. A bright-field image of individual δ-cells inside an Eppendorf cup, indicated by asterisks, before dissolving into solution as acquired by LMD7. The proteomes (top) and transcriptomes (bottom) of different single cell-containing solutions were analyzed.

As the processing time must be short, it is valuable to have a high islet density in the processed sample. Therefore, we analyzed islet concentrations in different mouse strains^32^. Immunohistological samples from C57BL/6 mice presented a few islets per slide. Moreover, the islets of C57BL/6 mice are relatively small, increasing the number of slides needed to obtain a sufficient amount of endocrine cells for analysis. Therefore, while achievable in the C57BL/6 background, we turned to Swiss Webster (CFW) mice, which have large islets with a high pancreatic density that allows faster islet cell collection for proving the methodological concept^33^ (Fig. 2a–c).

Pancreata were harvested from CFW mice, and endocrine α-, β- and δ-cells were isolated from rapidly immunostained slides using LMD (Fig. 2d,e). These cells were then analyzed for transcriptome and proteome characteristics (Fig. 2e).

### Proteomics

OCT is a resin mixture, protected by a patent, and its exact composition is not publicly disclosed. OCT is known to interfere with proteomic measurements using MS^34–36^. Therefore, we needed to find a matrix that: (1) is composed of a simple molecular mixture with similar physical properties to OCT and is compatible with MS measurements and (2) can maintain a solid encapsulation of the pancreas, be cut in the cryostat with standard blades without cracking or breaking the block and adhere to the Leica’s Director slides.

OCT block-making technology is a relatively new methodology and is currently the gold standard in histology. However, before its development, other techniques were used to keep biological samples in a block. Preceding OCT, different natural resins were used^37,38^. These natural resins also contain polymers of various lengths, which interfere with LC and ionize easily, thereby substantially disrupting LC–MS measurements. Therefore, we sought to create a semicrystalline block made from pure sucrose in water, which in MS creates a single peak for sucrose. To our gratification, this attempt was successful and the use of pancreata immersed in sucrose blocks yielded satisfactory results.

**Fig. 3:**
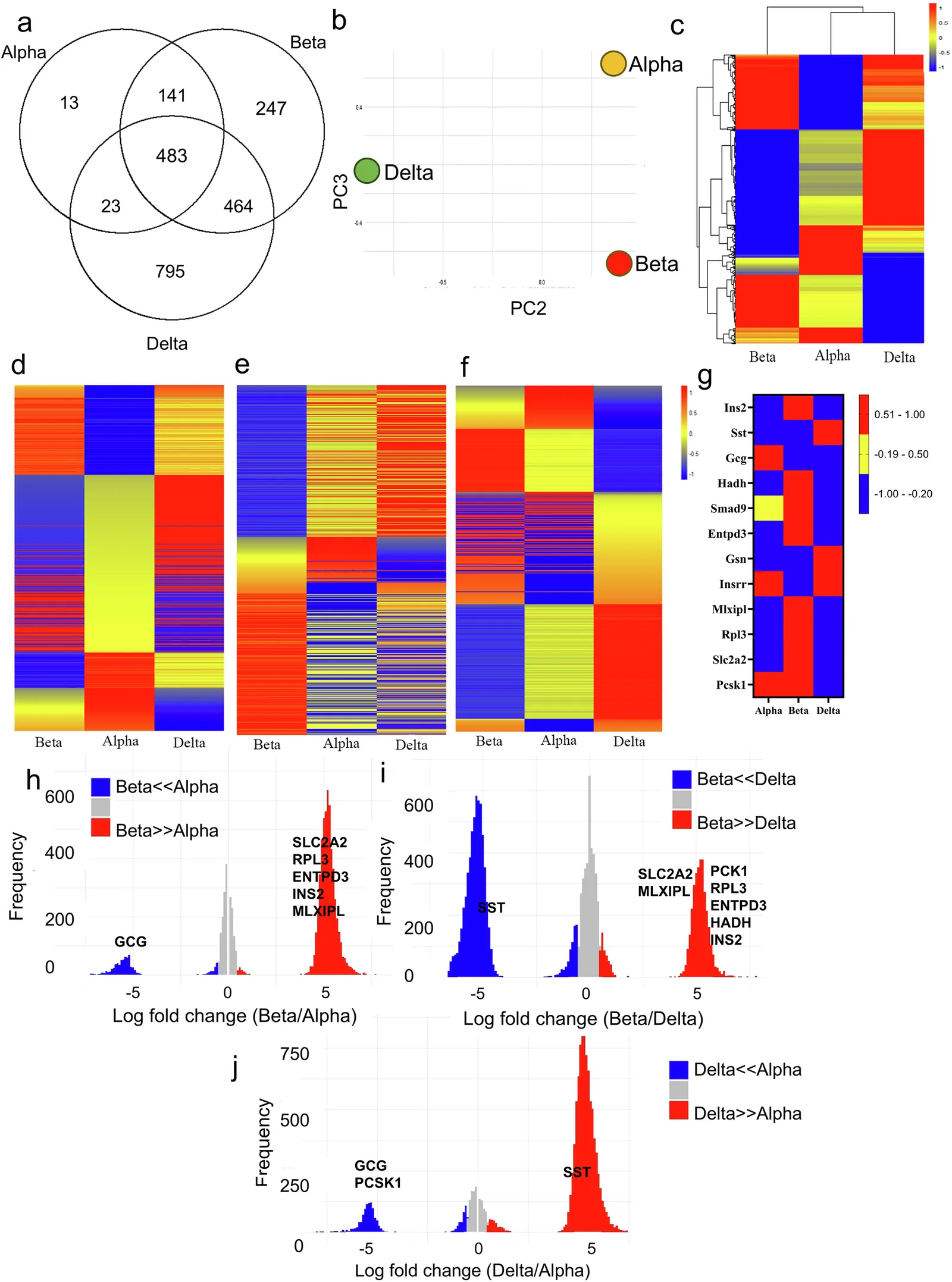
Proteomic data for the different endocrine cell types. **a**, Venn diagram of the different proteins identified according to the α-, β- and δ-cell types. **b**, PCA of the different cell types characterized according to their proteomic analysis. **c**, Heat map and corresponding dendrograms generated by the proteomic analysis. Note that α- and δ-cells are closer to each other than β-cells. **d**–**f**, Heat maps arranged by protein expression levels, sorted according to α-cells (**d**), β-cells (**e**) and δ-cells (**f**). Note that after this arrangement, β- and δ-cells appear as negatives of each other. **g**, High-profile proteins known to be specifically expressed in the different cell types are presented. **h**–**j**, Histogram analyses comparing α- and β-cells (**h**), β- and δ-cells (**i**) and α- and δ-cells (**j**). Some high-profile proteins are specifically indicated. For all panels, *n* = 6.

Blocks were sectioned and mounted on Director slides, designated for LMD-based proteomic studies. Individual α-, β- and δ-cells were collected into separate vials according to their cell type for proteomic analysis (Fig. 2d,e and Supplementary Movies 3, 4 and 5). The timsTOF Pro MS, operated in high-sensitivity mode, identified 660 proteins in α-cells, 1,335 in β-cells and 1,765 in δ-cells (Supplementary Excel Sheet 1). A Venn diagram (Fig. 3a) shows that 13 proteins were uniquely detected in α-cells, 247 in β-cells and 795 in δ-cells, indicating clear molecular distinctions among the cell types.

Principal component analysis (PCA) supported this separation, confirming that the three endocrine cell types form distinct molecular clusters (Fig. 3b). Heat-map analysis further validated these findings, with the dendrogram showing that α- and δ-cells are more closely related to each other than to β-cells (Fig. 3c). This clustering pattern may initially appear inconsistent with pancreatic developmental studies, which indicate that α-cells diverge earlier from the β- and δ-cell lineage, which arises from a shared progenitor^39,40^. To address this apparent contradiction, we reconstructed heat maps sorted by each cell type (Fig. 3d–f). Despite all samples originating from pancreatic islet tissue, and thus expected to express many common proteins, the expression patterns revealed a striking ‘negative’ relationship between β- and δ-cells—proteins highly expressed in one were often underexpressed in the other. This reciprocal pattern may explain the dendrogram’s classification, positioning β- and δ-cells far apart. One plausible explanation is differential transcriptional regulation. For instance, MLXIPL, a transcription factor identified in β-cells in our proteomic studies, may drive this divergence. Its expression correlates with key β-cell functions described in transcriptomic studies^19,41–43^.

We identified proteins that were uniquely expressed in each cell type, consistent with published transcriptomic profiles^19,44–51^ (Fig. 3g). As expected, α-cells showed high expression of glucagon (Gcg), β-cells of insulin (Ins2) and δ-cells of SST—the markers used to identify these cell types. In addition to these expected markers, several other proteins were detected that are known to be consistently expressed in these cell types in prior transcriptomic studies^19,44–51^. These findings reinforce the accuracy of our cell-type-specific isolation and proteomic identification, supporting that the cells were correctly classified by type.

Finally, we compared the cell-specific proteomic data. Histogram analysis indicated that the isolated populations exhibited more type-specific proteins than shared ones. The α-cells showed the fewest uniquely expressed proteins (Fig. 3h,j, blue), consistent with previous findings suggesting a relatively low-expression phenotype in this cell type^19,44–51^. Among these are, as expected, the Gcg and PCSK1 proteins. β-cells exhibited a broad set of unique proteins (Fig. 3h,i, red), including Ins2, SLC2A2, RPL3, ENTPD3 and MLXIPL—all of which have been previously reported in transcriptomic studies^19,44–51^. Surprisingly, δ-cells, which have historically received comparatively less focus in islet biology, exhibited the highest number of uniquely expressed proteins (Fig. 3i, blue, and Fig. 3j, red). As these cells remain less extensively characterized, only SST is specifically highlighted.

### Transcriptomics

**Fig. 4:**
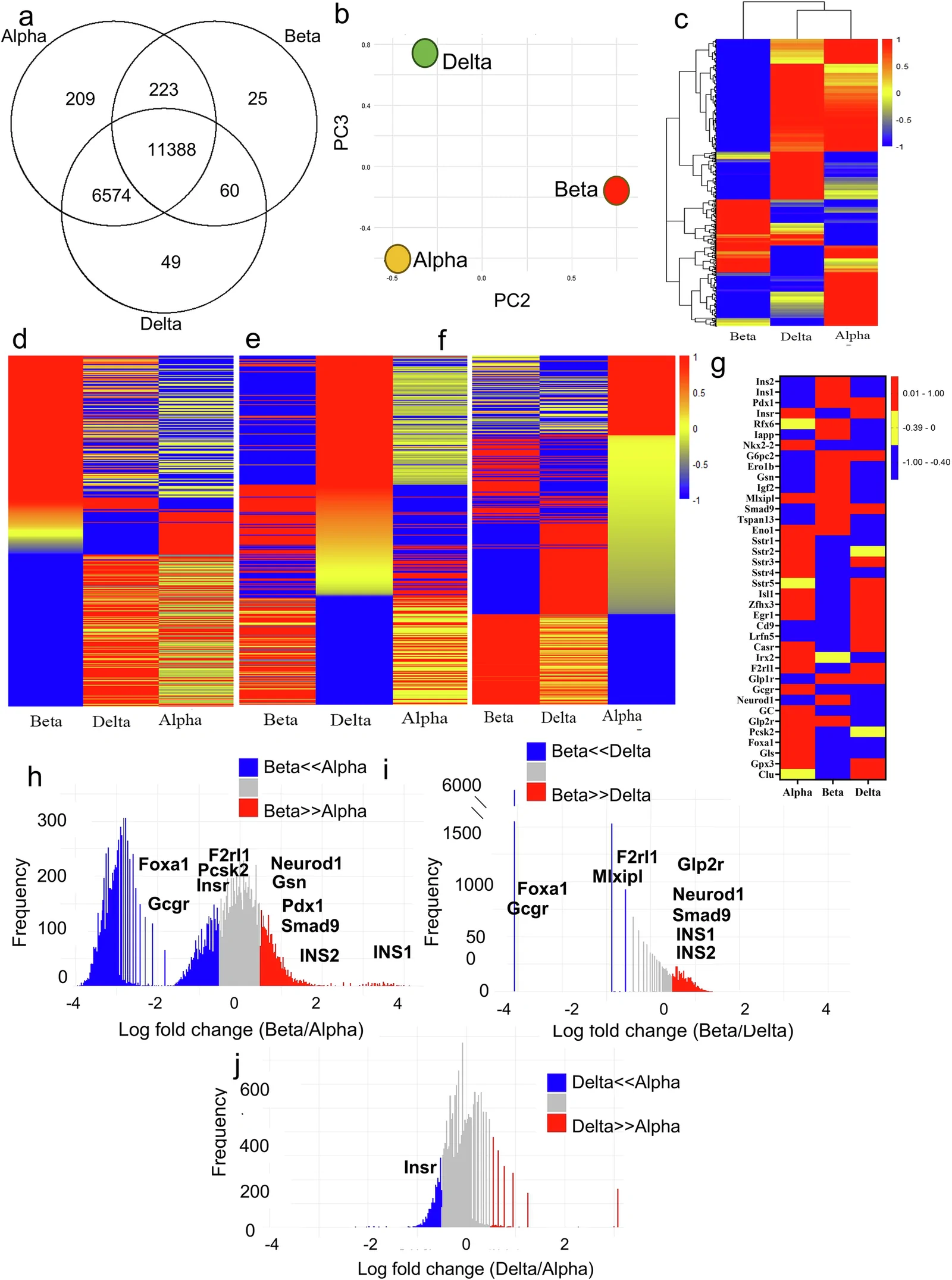
Transcriptomic data for the different endocrine cell types. **a**, Venn diagram of the different genes identified according to the α-, β- and δ-cell types. **b**, PCA of the different cell types characterized according to transcriptomic analysis. **c**, Heat map and corresponding dendrograms generated by the transcriptomic analysis. Note that, as in the proteomic analysis (Fig. 3), α- and δ-cells are closer to each other than β-cells. **d**–**f**, Heat maps arranged by gene expression levels, sorted according to α-cells (**d**), β-cells (**e**) and δ-cells (**f**). Note that after this arrangement, β- and δ-cells appear as negatives of each other. **g**, High-profile genes, known to be specifically expressed in the different cell types, are presented. **h**–**j**, Histogram analyses comparing α- and β-cells (**h**), β- and δ-cells (**i**) and α- and δ-cells (**j**). Some high-profile genes are specifically indicated. For all panels, *n* = 6.

LMD enables the acquisition of transcriptomic data as well. Here, OCT blocks did not interfere with the data collection. We used PET slides, which are characterized by a completely different cutting methodology (Supplementary Movies 6 and 7). Single-cell populations were analyzed by the Partek Flow, and 18,394 individual transcripts for α-cell mRNA, 11,696 individual transcripts for β-cells and 18,071 individual transcripts for δ-cells were identified (Supplementary Excel Sheet 2). A Venn diagram (Fig. 4a) was used to map 209 genes unique to α-cells, 25 unique to β-cells and 49 unique to δ-cells.

PCA indicated that the α-, β- and δ-cells were distinct populations, with a similar separation between the three cell types (Fig. 4b). Heat-map analysis confirmed the PCA results, and similar to the proteomic data (Fig. 3c), its dendrogram indicates that α- and δ-cells are closer to each other than to β-cells (Fig. 4c). According to our transcriptomic data, β- and δ-cells presented opposite profiles, albeit less apparent than in the proteomic data. The heat maps according to cell type (Fig. 4d–f) highlight this observation. In addition to the MLXIPL transcription factor protein, which we identified by proteomic analysis, we identified the Rfx6, Isl1, Zfhx3, Egr1 and NeuroD1 transcription factors that are differentially expressed between β-cells and δ-cells. Figure 4g shows high-profile genes that were also reported in other works^19,44–51^. These findings support the possibility that the isolated islet cells were from the indicated cell type. We compared the cell-specific transcriptomic data. As with the proteomics data, we found distinctive previously reported RNA transcripts indicative of the different cell types. Distinctive RNA transcripts of α-cells are shown in blue in Fig. 4h,j, and distinctive RNA transcripts of β-cells are shown in red in Fig. 4h,i. Distinctive RNA transcripts of δ-cells are shown in blue in Fig. 4i and in red in Fig. 4j. These transcripts correspond with previous findings^19,44–51^.

### Proteomic and transcriptomic data comparison

**Fig. 5:**
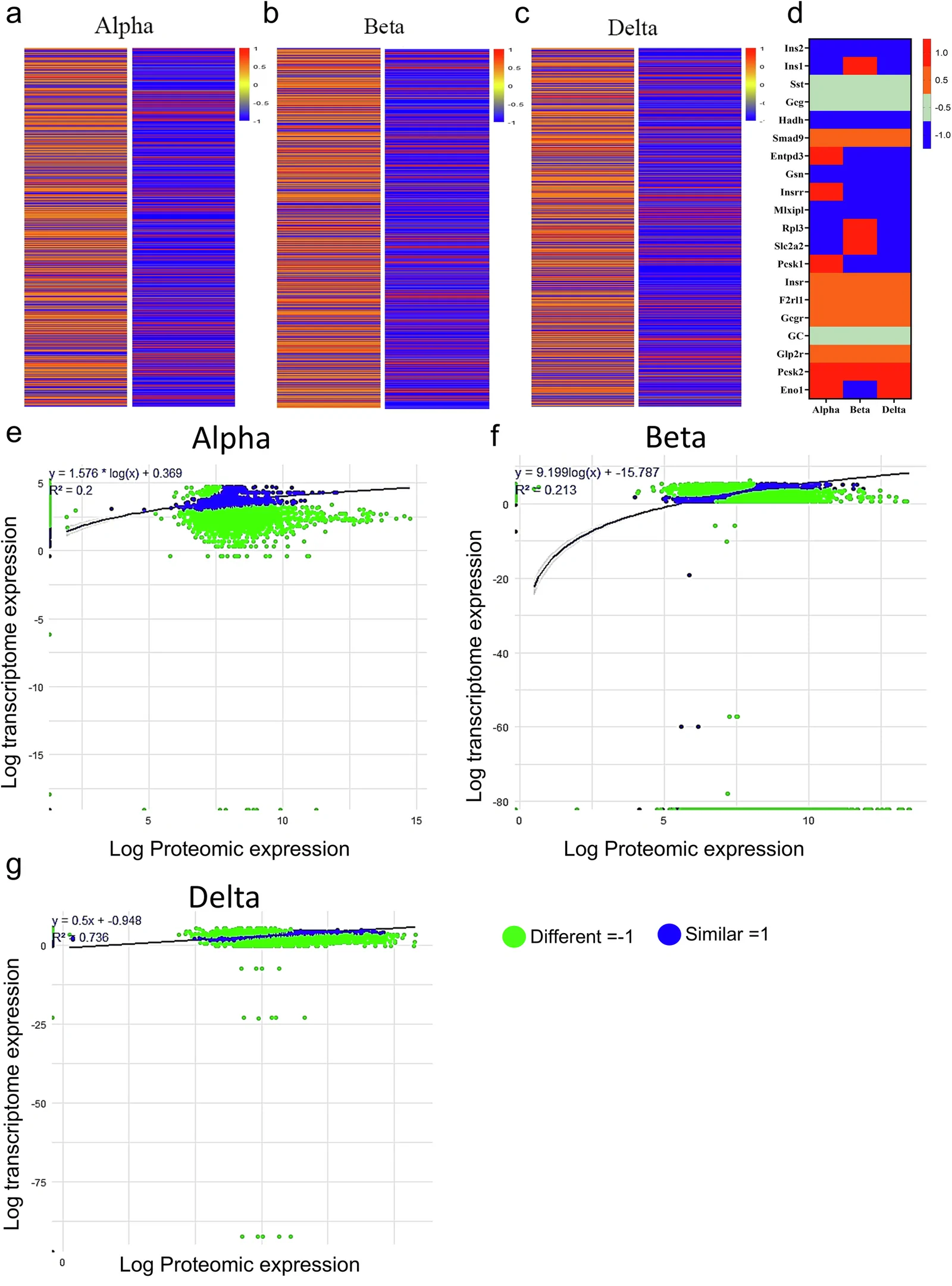
A comparison of the data achieved by different methodologies. **a**–**c**, Comparison of our proteomic data to our transcriptomic data according to α-cells (**a**), β-cells (**b**) and δ-cells (**c**). Relative values were compared, and values with a maximal difference of |0.15| were considered ‘similar’. Similarly expressed transcripts and proteins are shown in red, while differentially expressed transcripts and proteins are shown in blue. Proteins that are not represented with transcripts are shown in celadon. As transcriptomic analysis identified more genes than proteomic analysis (shown in orange), for each cell type, we generated two comparisons: one showing these genes (left) and one not presenting them (right). Note that most comparisons are marked ‘different’, indicating that gene expression levels are not similar to the final translated levels. **d**, Comparison of high-profile genes and proteins according to the different cell types. **e**–**g**, Plots of the raw data from each methodology to assess whether similarities and differences were calculated appropriately. For each cell type, α-cells (**e**), β-cells (**f**) and δ-cells (**g**), proteomic and transcriptomic data were plotted. Similar genes and proteins are clustered around a trendline, indicating the appropriateness of the methodology (in blue) compared with different genes and proteins that do not cluster at all (in green).

Proteomic and transcriptomic data are acquired using different methodologies, and the results are presented in different units. To compare the data, we first allocated relative values to the dataset (Supplementary Excel Sheet 3). Analysis allocated the following values: 1 for genes (and proteins) that are ranked similarly in both datasets (indicated in red), 0.5 for genes present in the transcriptomic data but missing from the proteomic data (in orange), −0.5 for peptides present in the proteomic data but absent from the transcriptomic data (in celadon) and −1 for peptides and genes ranked differently in both datasets (in blue) (see the ‘Materials and methods’ section for elaboration; Fig. 5a–d). To check the validity of the data generated, we correlated the raw data of both methodologies and plotted them against each other (Fig. 5e–g). Similar genes and proteins that received the value 1 were clustered, surrounding a trendline (similar genes and proteins marked in blue), while different genes and proteins (−1 value), could not be clustered surrounding a defined trendline function (in green).

As RNA-seq is known to be more sensitive than proteomics, most of the genes had a value of 0.5, indicating that they were not identified in the proteomic data (Fig. 5a–c, left). To obtain more meaningful information, we omitted this value to create the right panels of these figures. From this comparison, we understand that proteomic and transcriptomic data obtained by the same researchers using similar methods still yield very different values, indicating that RNA levels do not always correspond with protein levels and highlighting the importance of complementing RNA-seq with proteomic data. Figure 5d shows the high-profile genes identified in the field.

### Comparing our data to literature

**Fig. 6:**
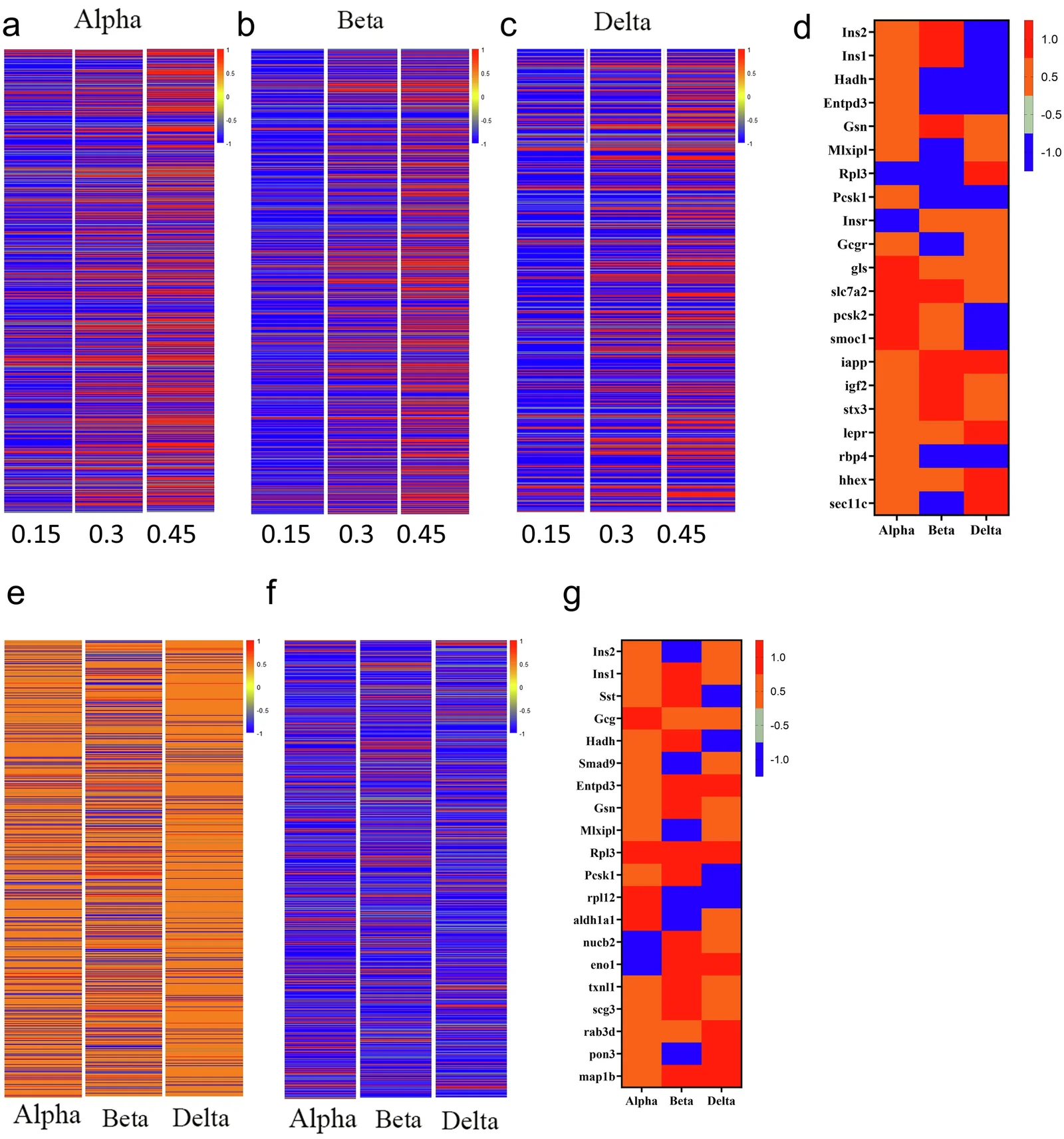
A comparison of our results to data achieved by proteolytic methods. **a**–**c**, Comparison of our transcriptomic data obtained by the mechanistic method to the compiled transcriptomic data obtained by a proteolytic method^19^. The data are shown for α-cells (**a**), β-cells (**b**) and δ-cells (**c**). Similarly expressed genes are shown in red, while differentially expressed genes are shown in blue. Genes that are not represented in our data are presented in celadon. As our data are larger than the compiled data, we moved the comparisons that included genes that appeared in our dataset and not in the compiled data to Supplementary Fig. 2a–c, respectively. In all our comparisons we defined a difference threshold of |0.15| as similar. Here, we show that threshold (left) and increase it to |0.3| (middle) and to |0.45| (right). Notably, in all presentations, there were significant numbers of genes that were differentially expressed, indicating that the mechanistic separation of the endocrine cells generated different data than what was achieved by enzymatic proteolysis. **d**, Comparison of high-profile genes obtained from our transcriptomic data to the compiled transcriptomic data received by a proteolytic method according to the different cell types. Similarly expressed genes are shown in red, while differentially expressed genes are shown in blue. Genes that are not represented in the compiled enzymatic data are presented in orange. **e**,**f**, Our proteomic data were compared with the data obtained by Van Gurp et al.^19^. Similarly expressed proteins and Van Gurp’s transcripts are shown in red, while differentially expressed proteins and Van Gurp’s transcripts are shown in blue. Data appearing only in Van Gurp’s transcripts are shown in celadon. Proteins found by our analysis that do not have a transcriptomic counterpart are shown in orange. **e**, Representation of all the data. **f**, Representation of the data with omission of the incomparable proteomic data (orange in **e**). **g**, Comparison of high-profile genes obtained from our proteomic data to the compiled transcriptomic data according to the different cell types. Similarly expressed genes and proteins are shown in red, while differentially expressed genes are shown in blue. Proteins that are not represented in the compiled transcriptomic data are presented in orange.

In their meta-analytic study, van Gurp et al. compiled recent literature on transcriptomic analysis in pancreatic islets, all obtained by single-cell enzymatic separation^19^. This seminal work provides a good baseline for comparing data. The advantage of their work is that it averages diverse data and highlights the genes that are significant and consistently reported across different studies. However, this approach also omits many genes that are either not significant or not repeated in these studies, resulting in a relatively short list of genes (862 for α-cells, 1,870 for β-cells and 647 for δ-cells)^19^. Our analysis, however, included many more genes than this meta-analysis.

van Gurp et al. used relative units ranging from 1 (least abundant) to 21 (most abundant), as they compared different studies to generate a 21-ladder scale. Thus, we transformed their transcriptomic data into our scale (Supplementary Excel Sheet 4). As described above, we allocated the following values: 1 for genes ranked similarly in both datasets (shown in red), 0.5 for genes present in our data but missing from the van Gurp et al. data (in orange), −0.5 for genes present in the van Gurp et al. data but absent from our transcriptomic data (in celadon) and −1 for genes ranked differently in both datasets (in blue; Supplementary Excel Sheet 5; see the ‘Materials and methods’ section for elaboration). Figure 6a–c does not show the 0.5 data, which are shown in Supplementary Fig. 2a–c, respectively.

Note that for each of the heat maps in Fig. 6a–c, there are three columns. The prominent colors in the left columns are blue (−1, different). However, as we move to the right, the abundance of the red color intensifies. This is to explain our methodology. We used a stringent policy, in which we limited the indication of ‘similar’ to a difference of no more than |0.15| on the 21-ladder scale described above. As we allowed a greater threshold, the genes became more similar (indicated by increasing ‘1’ values in red). The middle columns use a |0.3| threshold, and the right columns use a |0.45| threshold.

Even when using the |0.45| threshold difference, many of the genes were ‘different’ (in blue), demonstrating the influence of the cell isolation methodology on the obtained results, as the van Gurp et al. data were obtained from cells dispersed by proteolytic cleavage (mostly for FACS analysis), while our data were generated from direct nonproteolytic isolation from slides using LMD. Figure 6d shows ‘high-profile’ genes using the |0.15| threshold.

We also conducted a similar comparison of the van Gurp et al. data to our proteomic data (Fig. 6e–g, scaling of the proteomic data from the 0 (most abundant)-to-1 (least abundant) scale; Supplementary Excel Sheet 6). Figure 6e shows all genes and proteins. In Fig. 6f, we omitted the peptides that are missing from the van Gurp et al. dataset (0.5 value).

As β-cells are the most studied cell type, we found the highest correspondence in between the two datasets. Interestingly, even for β-cells, we found many ‘differently’ expressed genes, showcasing once more the difference between transcription and translation.

Recently, Thielert et al. reported a single-cell proteomic study characterizing α-, β- and δ-cells using FACS, as part of a broader analysis comparing proteomics and phosphoproteomics under healthy and inflammatory conditions^23^. This important work provides a reference point for comparing enzymatically based FACS and mechanistic isolation techniques. The Thielert et al. study encompasses more than 6000 proteins per cell type; however, not all protein readouts were consistent. Therefore, for downstream analysis, we included only proteins displaying a standard deviation ≤0.25 (Supplementary Excel Sheet 7). We applied the same 1, 0.5, −0.5 and −1 indexing scheme described above, using the stringent |0.15| threshold (Supplementary Fig. 2d–f).

As our dataset is larger than the processed Thielert et al. dataset, most proteins in the comparison are classified as 0.5 (orange) (Supplementary Fig. 2d). However, even after excluding the 0.5-marked proteins to generate Supplementary Fig. 2f, most remaining proteins are marked in blue, representing a ‘different’ value (−1). Notably, the methodological differences yielded the greatest divergence in α-cells, whereas β-cells exhibited the largest number of ‘similar’ proteins, represented in red.

### Differentially expressed genes and proteins

Our methodology identified numerous differentially expressed genes and proteins, prompting us to characterize some of the most abundantly expressed among them. As reflected by the dominance of orange in Fig. 6e and Supplementary Fig. 2a–d, many of the gene products we identified were newly associated with specific islet cell types, being present in our dataset but absent from both the van Gurp et al. and the Thielert et al. datasets. Given the size of these datasets, fully provided in Supplementary Excel Sheets 1–7, we could not discuss all findings in detail. Instead, we focused on the most highly expressed genes and proteins within each cell type that also show functional relationships as part of coherent biochemical pathways, as revealed by STRING analysis^52^.

Importantly, the identification of gene products and pathways enriched in one cell type does not imply absence from others but rather reflects relative expression, where such pathways may be outcompeted by other, more highly expressed gene products in the second cell type. A notable finding emerging from this analysis was the prominence of membrane-associated pathways across all endocrine cell populations. Rather than functioning solely as hormone-producing cells, the molecular profiles suggest that pancreatic endocrine cells possess specialized systems dedicated to environmental sensing, intercellular communication, signal integration and regulation of tissue-level responses. Collectively, these findings support a view of the islet as an integrated sensory and regulatory network, in which endocrine cells actively monitor and respond to signals originating from neighboring cells and the surrounding microenvironment. From this perspective, hormone secretion represents only one component of a broader biological program involving environmental sensing, signal processing and coordinated multicellular responses. While transcriptomic analyses, as reflected in the compiled van Gurp et al. study, did not highlight membranal proteins, proteomic studies, reflected in both the Thielert et al. study and our results, identified membranal protein populations.

**Fig. 7:**
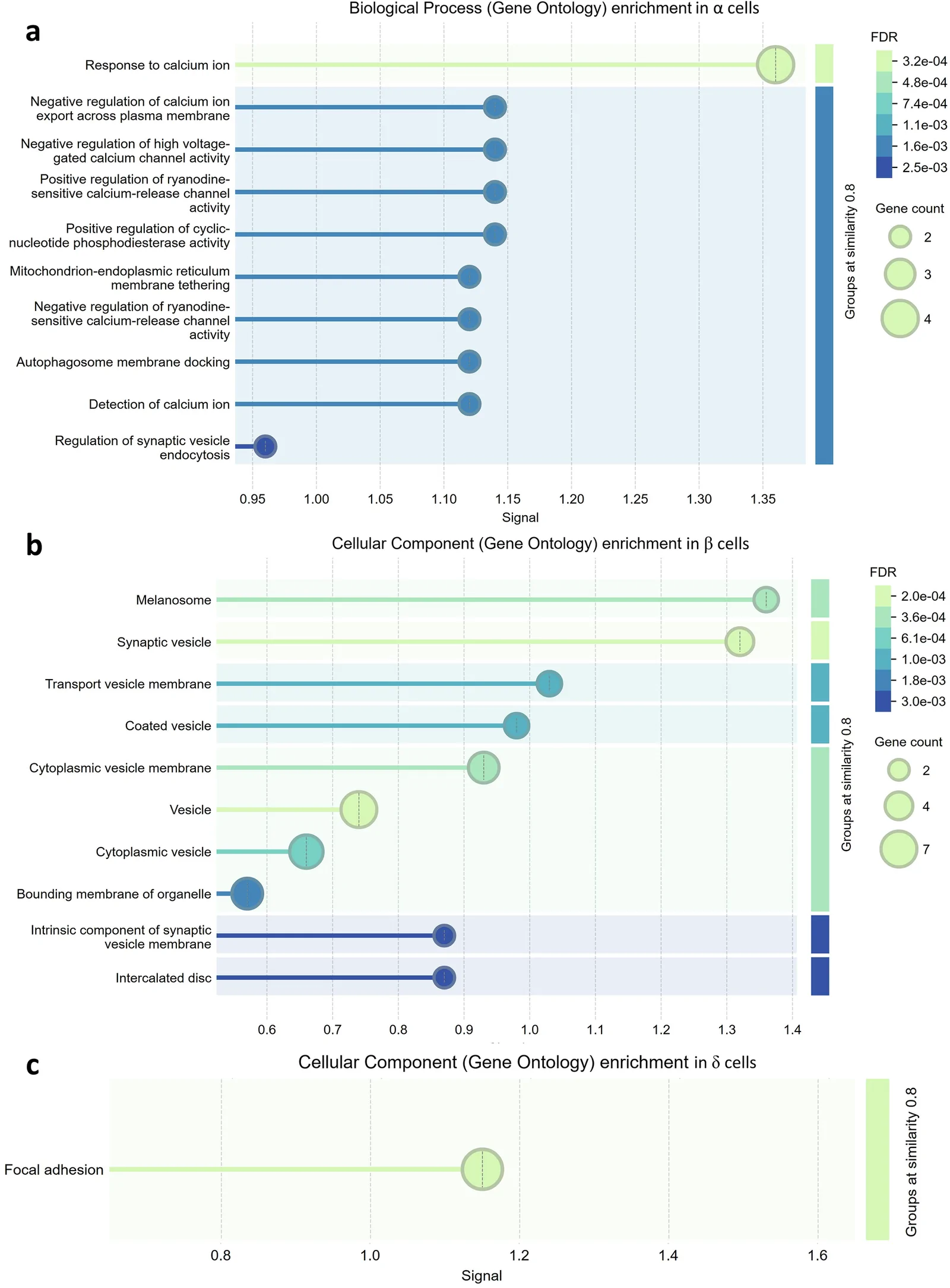
Distinctive pathways characterized by ‘different’ genes identified in our nonenzymatic dataset in α-, β- and δ-cells. **a**–**c**, Genes and proteins identified as ‘different’ were ranked by their expression levels for α- (**a**), β- (**b**) and δ-cells (**c**), excluding general housekeeping genes. STRING analysis was performed using default settings^51^. In **a**, α-cells are enriched for genes and proteins involved in action potential regulation, particularly calcium signaling. In **b**, β-cells exhibit a high expression of genes and proteins related to vesicle trafficking pathways. In **c**, δ-cells show significant expression of genes and proteins associated with focal adhesion-related pathways.

α-cells were found to highly express genes and proteins involved in action potential regulation, particularly calcium-related pathways, as indicated by STRING analysis of our dataset (Fig. 7a; see Supplementary Fig. 3a for a detailed presentation). These pathways are consistent with the central role of α-cells in sensing metabolic fluctuations and coordinating glucagon secretion in response to changing physiological demands^53,54^. β-cells exhibited high expression of vesicle trafficking-related genes and proteins (Fig. 7b; see Supplementary Fig. 3c for a detailed presentation). Given the dependence of insulin secretion on tightly regulated vesicle mobilization and exocytosis, the enrichment of these pathways supports the biological relevance of the identified β-cell signatures^55,56^. δ-cells showed significant expression of focal adhesion-related genes and proteins (Fig. 7c; see Supplementary Fig. 3b for a detailed presentation). The prominence of these pathways suggests an active role for δ-cells in mediating interactions with neighboring endocrine cells and the surrounding microenvironment, potentially contributing to the regulatory functions of SST within the islet. Thus, our analysis further organized these proteins into coherent functional pathways, enabling biological interpretation beyond individual molecular markers.

## Discussion

The major contribution of this work is the generation of proteomic profiles of pancreatic endocrine cell types using a mechanistic single-cell isolation technique.

Characterizing pancreatic tissue presents unique challenges. The pancreatic exocrine gland expresses digestive enzymes^57,58^, which break down RNA and proteins into their primary building blocks, such as individual nucleotides and amino acids. This enzymatic activity can destroy proteomic and transcriptomic data, reducing it to a primordial soup. Therefore, a quick and delicate approach is required to isolate samples before they are enzymatically destroyed. Importantly, in the digestive biochemical pathway, pancreatic zymogens are activated by trypsin, and a single trypsin molecule can activate thousands of zymogens^59,60^. Thus, approaching the pancreatic tissue with trypsin necessitates extra care.

In addition, the pancreatic islet is a heterogeneous environment, harboring different cell types closely packed together in an unorganized form, creating a mixed mosaic of cells. Traditionally, single-cell studies have relied on enzymatic–proteolytic dissociation to isolate cells, but this process can compromise membrane proteins. In subsequent stages, the dispersed cells were separated into subpopulations. The preliminary enzymatic stage can lead to the loss of external proteins, resulting in their exclusion from the dataset. Moreover, the presence of enzymatically activated receptors, such as protease-activated receptors including Par2, can pose challenges, as Par2 activation changes gene expression^25^.

In this work, we describe an alternative approach in which tissue is fresh frozen before single-cell isolation; individual endocrine cell types are collected by mechanistic, nonenzymatic LMD rather than enzymatic dissociation, yielding cell-type-specific proteomic and transcriptomic profiles. The first aim of this work was to establish that LMD can be adapted for reliable isolation of defined endocrine cell populations from the highly heterogeneous environment of the pancreatic islet. Achieving this goal required overcoming several technical challenges, including the development of a rapid immunostaining workflow compatible with molecular preservation, elimination of conventional fixation procedures and the implementation of sucrose-based embedding conditions compatible with downstream MS. Once this was achieved, a subsequent aim was to determine whether islet cell characterization differs between mechanistic and proteolytic separation. Unlike conventional enzymatic workflows, the present approach preserves tissue architecture, avoids proteolytic disruption of membrane-associated proteins and minimizes isolation-induced alterations in gene expression. Although fixation methods such as PFA can improve histological resolution, our findings indicate that chemical fixation impairs molecular yield and data quality in both proteomic and transcriptomic analyses. Therefore, we used a rapid fresh-freezing protocol to better preserve the native molecular state of the islets for accurate single-cell profiling.

Our use of a nonenzymatic approach for isolating individual islet cells is not without precedent. Bengtsson et al. previously used guanidine thiocyanate as a chemical dispersal agent to separate islet cells, enabling the isolation of approximately 20–30 pancreatic β-cells for targeted mRNA analysis using reverse transcription PCR (RT–PCR)^61,62^. However, that approach was designed to determine whether specific transcripts were present in β-cells, rather than to comprehensively characterize the full transcriptomic or proteomic profile of individual islet cell types. By contrast, answering the broader question of what islet cells express requires substantially larger cell numbers. Approximately 400 cells were collected into each Eppendorf cap in our workflow, representing a cell number more than an order of magnitude higher than that used by Bengtsson et al. (‘Materials and methods’ section). This number approached the sensitivity limits of both the timsTOF Pro MS/MS and Illumina NextSeq 500 platforms. We anticipate that as single-cell omics technologies continue to advance in sensitivity, future studies may successfully revisit and expand on early chemical dispersion methods using modern high-resolution instrumentation.

Recent studies examining individual profiles of islet cells indicate that the spatial location of a cell within the islet may influence its function and, therefore, the genes and proteins it expresses^9,10^. Moreover, studies highlight the important role of intra-islet communication between islet cell types in regulating their function^11^. While in this preliminary work we focused on demonstrating the suitability of LMD technology for studying islet cell omics, future efforts may utilize LMD’s ability to isolate a particular cell on the basis of spatial positioning, pool similarly located cells and profile their gene products according to their spatial arrangement. This feature is of special interest in pathology, where diabetes and related disorders may alter islet microarchitecture and disrupt cell–cell communication.

There was a challenge in comparing the proteomic and transcriptomic results, as these are achieved differently, with different methodologies. In addition, the sizes of the data achieved are also different for each technique. There are a few ways to try and compare such data. One is to assign a minimal score to the most abundant result and continue to the end of the smaller dataset, truncating the larger dataset accordingly and continuing the scoring from that point. Another way is to apply the same normalization treatment to each dataset, regardless of size. We tested both strategies and found the latter to better reflect reality, as ‘similar’ genes and proteins clustered around a trendline for all cell types (Fig. 5e–g).

The recent work by Thielert et al. demonstrated that proteomic characterization of pancreatic endocrine cell types can be achieved using FACS-based isolation^23^. This pioneering study provided an opportunity to directly compare proteomic datasets generated by enzymatic and mechanistic cell separation techniques. Such a comparison is shown in Supplementary Fig. 2d–f. The analysis demonstrates a strong influence of the isolation methodology on the resulting molecular readouts. The majority of proteins were scored as different, with α-cell populations showing the greatest variability, indicating that relative expression levels are highly sensitive to the method of cell isolation. We hypothesize that this effect is mediated primarily by Par2, which is activated by proteolytic cleavage. Definitive testing^24,26,27,63^ of this hypothesis would require comparative FACS-based islet proteomic analyses in Par2 knockout and wild-type mice.

As our methods enable the generation of transcriptomic data, we generated and compared our data with literature. The van Gurp et al. study was instrumental and compiled significant data from trypsin and trypsin-like isolated pancreatic endocrine islet cells, mostly separated by FACS, into a comprehensive meta-analytic study^19^. Our results indicate that while the data are generally comparable, many genes are expressed differently (Fig. 6a–c).

The fact that many ‘different’ genes and proteins in our mechanistic dataset encode membrane-associated proteins is noteworthy, as enzymatic methods such as FACS can strip membrane proteins during cell preparation. However, it is important to note that enzymatic isolation generates RNA-seq data, and coding RNA molecules are neither located on membrane surfaces nor directly accessible to proteolytic degradation. Therefore, we do not yet have a clear hypothesis as to why our method preferentially identified membrane-associated proteins as differentially expressed.

Beyond their technical recovery, the biological significance of these proteins lies in their central role in how endocrine cells sense, interpret and respond to their microenvironment. Membrane-associated proteins mediate nutrient sensing, hormone responsiveness, electrical activity, cell–cell communication and interactions with the extracellular matrix. Consequently, changes in these pathways have the potential to influence both endocrine cell identity and islet-level coordination.

Nevertheless, because many membrane proteins serve as disease biomarkers and therapeutic targets, their preservation has particular significance for endocrine pathology. Interestingly, in the proteomic characterization reported by Thielert et al., the authors identified several membrane-associated proteins, suggesting that limitations in detecting such proteins may be linked more to constraints of RNA-seq methodologies than to proteolytic cell dissociation per se. Specifically, they reported membrane-associated proteins in α-cells, including Ace2, Hapln1 and Hk1; in β-cells, proteins such as Emilin1, Rtn4 and Wls; and in δ-cells, proteins including Gap43, Ncam2 and Spock1^23^. However, these findings were not further clustered or mapped to defined biochemical pathways.

A distinction of the present study is that these proteins were not only detected but also organized into coherent pathway-level signatures characteristic of each endocrine cell type. This organization enabled biological interpretation of membrane-associated processes and revealed functional specialization across α-, β- and δ-cells that was not readily apparent from individual protein measurements alone.

The finding of calcium-dependent action potential regulation in α-cells in our dataset aligns with their known physiology, where calcium influx plays a crucial role in triggering glucagon secretion^64^. Unlike β-cells, which are stimulated by glucose, α-cells are inhibited by high glucose concentrations through a glucose/ATP-dependent mechanism^53^. The regulation of glucagon secretion involves voltage-gated ion channels that control calcium dynamics, as α-cells exhibit spontaneous electrical activity at low glucose levels, leading to calcium influx and glucagon release^54^. Further studies have highlighted that α-cells rely on precise electrophysiological mechanisms, including ATP-sensitive K^+^ (K_ATP) channels, to regulate intracellular calcium oscillations and coordinate glucagon secretion^65^. Our findings reinforce the molecular underpinnings of these processes, suggesting that α-cells have a specialized action potential machinery tailored to their unique metabolic function and providing a molecular framework that may be altered under diabetic pathology.

The relatively elevated expression of calcium-dependent action potential mechanisms in α-cells does not imply their absence in β-cells, where these pathways are also well established^66^. For example, although Calm1 and Calm3 were prominently expressed in α-cells, they were also detected in β-cells, ranking 426 and 649, respectively—below our self-imposed threshold for defining functional pathway enrichment (Supplementary Fig. 3a). Anxa5, which appeared highly in the α-cell dataset (ranked 155), was also relatively high in β-cells (ranked 200); however, its associated action potential components were ranked much lower in β-cells. As a result, Anxa5 did not cluster with related genes to form a defined module and thus did not indicate the presence of a specialized action potential machinery in β-cells. These findings do not contradict the well-established role of calcium and ion channel activity in β-cell physiology^55^ but instead reflect the relative enrichment and coherence of these pathways in α-cells under our ranking-based analytical approach.

It is also not surprising that we identified vesicle trafficking mechanism-related genes and proteins highly expressed in β-cells, as pancreatic β-cells are specialized for insulin storage and regulated secretion, requiring a highly developed vesicle trafficking system^67^. Previous studies have highlighted the abundance and diversity of vesicles in β-cells, including large dense-core vesicles for insulin storage and synaptic-like microvesicles involved in exocytosis and intercellular communication^56^. In addition, β-cells exhibit dynamic vesicle recruitment mechanisms that facilitate glucose-dependent insulin release^68^. While these findings underscore the significance of vesicle trafficking in β-cells, direct comparative analyses with other pancreatic endocrine cells, such as α-cells and δ-cells, remain limited. Given our identification of vesicle trafficking-related genes and proteins highly expressed in β-cells, our findings suggest a need for quantitative studies comparing the vesicle content and volume across different pancreatic endocrine cell types. Such comparisons could be particularly informative in diabetes, where secretory capacity is compromised.

Our proteomic and transcriptomic analyses further revealed that δ-cells exhibit significantly higher expression of focal adhesion-related genes and proteins than α- and β-cells. This may be explained by the unique morphology of δ-cells, which extend long cytoplasmic projections to establish extensive contact with neighboring α- and β-cells. These interactions allow δ-cells to exert paracrine control over insulin and glucagon secretion, as previously suggested in studies highlighting their role as islet modulators^69^. Notably, recent high-resolution imaging work demonstrated that δ-cells use specialized filopodia-like projections to interact dynamically with multiple islet cells^70^. However, the molecular basis of this structural adaptation remains largely unexplored. Our findings provide a molecular signature supporting this functional specialization and suggest that δ-cells rely on focal adhesion mechanisms to maintain these intercellular contacts. Given limited literature on δ-cell morphology and function, future studies should systematically examine how these structural features influence islet dynamics and metabolic regulation. This may have relevance for understanding how δ-cell function is disrupted in pathological states, such as diabetes. The present findings should be interpreted within the scope of the experimental design. The study was performed under physiological conditions and was intended to establish reference molecular profiles of pancreatic endocrine cell types while identifying biological signatures revealed by mechanistic isolation. Accordingly, the data provide insight into endocrine cell identity and function in the healthy islet but do not directly address disease-associated remodeling of these pathways. Investigation of such changes will require dedicated studies using appropriate disease models and strain-matched controls, enabling direct assessment of molecular alterations associated with pathological states.

The lack of a direct relationship between RNA transcripts and the resulting protein levels was also demonstrated here^22,71^. Our results support this observation, showing substantial differences between proteomic and transcriptomic data (Fig. 5a–d). Moreover, in our proteomic analysis, α-cells yielded substantially fewer detectable proteins than β- and δ-cells (Fig. 3a), despite undergoing the same tissue preparation, isolation and MS workflow. This result contrasts with our transcriptomic data, where α-cells expressed transcript numbers comparable to δ-cells and significantly higher than β-cells (Fig. 4a).

This discrepancy suggests that α-cells may exhibit lower global protein synthesis rates or enhanced protein turnover, leading to a reduced steady-state proteome despite an active transcriptome. Alternatively, α-cells may exert tighter post-transcriptional control, limiting translation or stabilizing only a subset of transcripts. These findings highlight the importance of multi-omic profiling and suggest that α-cells, while transcriptionally active, may adopt a more restrained translational program compared with their islet counterparts. As proteins perform cellular functions, obtaining direct proteomic data is essential for understanding cellular functions, mechanisms and, in the case of the islets of Langerhans, for identifying molecular alterations relevant to diabetes and other endocrine disorders.

Our approach highlights the importance of fresh freezing the cells before enzymatic separation, as enzymatic dispersion and isolation may alter gene expression. This underscores the value of the nonenzymatic methodology presented in this work, which provides a more accurate and natural representation of cellular states without the influence of enzymatic processing.

One contribution of this study is the observation that CFW mice exhibit larger and denser islets than other commonly used strains. This property represents an added value of the strain, enabling higher yields during islet cell isolation and collection. For this reason, we chose to develop and optimize our isolation workflow using CFW mice. Now that the methodology has been established, it can be readily applied to other mouse strains, including NOD and Rcs-10 (NONcNZO10), which are widely used models for type I and type II diabetes, respectively^72–74^. In addition, the CFW strain is well established in metabolic research^33,75–77^ and has been used across a range of related fields^78–84^. Its genetic and physiological background differs from that of the inbred C57BL/6 strain, whose genetic uniformity can amplify strain-specific effects. In this context, the greater genetic diversity of CFW mice may help filter out background-dependent artifacts, allowing more robust and broadly generalizable observations to emerge.

Our technology physically separates individual cells according to their type, making it particularly relevant for heterogeneous tissues where cells are densely packed and intermixed, such as the endocrine islets of Langerhans. This approach complements established methods such as FACS, which remains the standard for high-throughput transcriptomic analysis. Each method therefore offers distinct advantages: while FACS excels in speed and scale, our workflow preserves native molecular states and captures proteomic features that may be compromised by enzymatic digestion.

Beginning with tissue fresh freezing provides a ‘snapshot’ of the tissue’s state at the time of fixation, yielding a pre-intervention view of the cells. This comes at the tradeoff of not capturing dynamic processes in living cells, which enzymatic approaches can sometimes reveal. Similarly, while our workflow requires manual identification and isolation of each cell—a time-intensive process—this careful handling contributes to the accuracy and fidelity of the proteomic data. By contrast, once cells are labeled, FACS can achieve high-throughput separation but often at the expense of membranal integrity.

While the current study does not yet achieve true single-cell resolution—requiring pooled captures of hundreds of cells per type to meet present MS detection thresholds—it nonetheless represents a critical step toward that goal. By resolving cell-type-specific proteomes while preserving native molecular states, our approach defines a practical benchmark on the path to true single-cell proteomics, which will become increasingly feasible as MS sensitivity continues to advance.

This study demonstrates that the method of cell isolation is not merely a technical consideration but a major determinant of the molecular profiles obtained from pancreatic islet cells. Enzymatic separation methods, such as FACS, are valuable for isolating live cells and have yielded important insights across many systems. However, as these approaches rely on enzymatic dissociation of living cells, from their native tissue environment, they may induce stress responses, activate signaling pathways, alter gene and protein expression programs and affect membrane-associated proteins. Our mechanistic LMD workflow instead uses fresh-frozen sections, preserving cellular architecture and molecular integrity at the time of tissue collection and providing a molecular snapshot that more closely reflects the in vivo state.

Importantly, the advantages of this approach extend beyond preservation alone. By avoiding enzymatic processing, we consistently recovered membrane-associated pathways that were diminished or absent in comparative enzyme-based datasets and identified molecular features that remained obscured using conventional workflows. These included membrane-associated proteins, signaling pathways and transcriptional regulators that contributed to cell-type-specific biological signatures. Thus, the mechanistic isolation strategy not only preserves molecular information but also expands the spectrum of detectable biological processes.

Beyond islet biology, the approach provides a broadly applicable framework for spatially resolved multi-omic analysis in cellularly heterogeneous and structurally complex tissues. As the approach preserves native tissue organization while minimizing isolation-induced perturbations, it may be particularly valuable in systems where cellular context and spatial relationships contribute significantly to biological function.

In summary, our findings highlight that the cell isolation strategy can substantially influence biological interpretation. While different methods each have advantages, enzyme-free LMD offers a powerful alternative when the preservation of native molecular states is paramount. We anticipate that its adoption will enhance the accuracy of spatial omics and expand opportunities for biological discovery across tissues and disease contexts. This framework may be applied in future work to comparative analyses of healthy and diabetic islets in mouse models, including nondiabetic and early diabetic NOD mice, as well as in human donor material such as nPOD samples. These studies would allow systematic assessment of disease-associated changes in islet cell biology and function.

## Supplementary information

Supplementary Information

Supplementary Movie 1. A movie showing specific excision of pancreatic β-cells (in blue).

Supplementary Movie 2. A movie showing the specific excision of pancreatic α-cells (in green).

Supplementary Movie 3. A movie showing the specific excision of pancreatic β-cells.

Supplementary Movie 4. A movie showing the specific excision of pancreatic α-cells.

Supplementary Movie 5. A movie showing dissected single δ-cells in the Eppendorf cup.

Supplementary Movie 6. A real-time movie showing the process of dissecting the different single cell types into different Eppendorf tubes.

Supplementary Movie 7. A real-time movie showing the process of dissecting the different single cell types into different Eppendorf tubes.

Supplementary Excel Sheet 1

Supplementary Excel Sheet 2

Supplementary Excel Sheet 3

Supplementary Excel Sheet 4

Supplementary Excel Sheet 5

Supplementary Excel Sheet 6

Supplementary Excel Sheet 7

## Data Availability

All data that support the findings of this study are available within this Article and its Supplementary Information or from the corresponding author on reasonable request.
