# Supplementary material for: Proteomic analysis of pancreatic endocrine cells by mechanistic single-cell isolation identifies membrane pathways
Source: Exp Mol Med. 2026 Sep 3;58(9):3007–24. doi: 10.1038/s12276-026-01826-9 (PMC13633244; doi:10.1038/s12276-026-01826-9)
Supplement: Supplementary file 1 [file 12276_2026_1826_MOESM1_ESM.pdf]

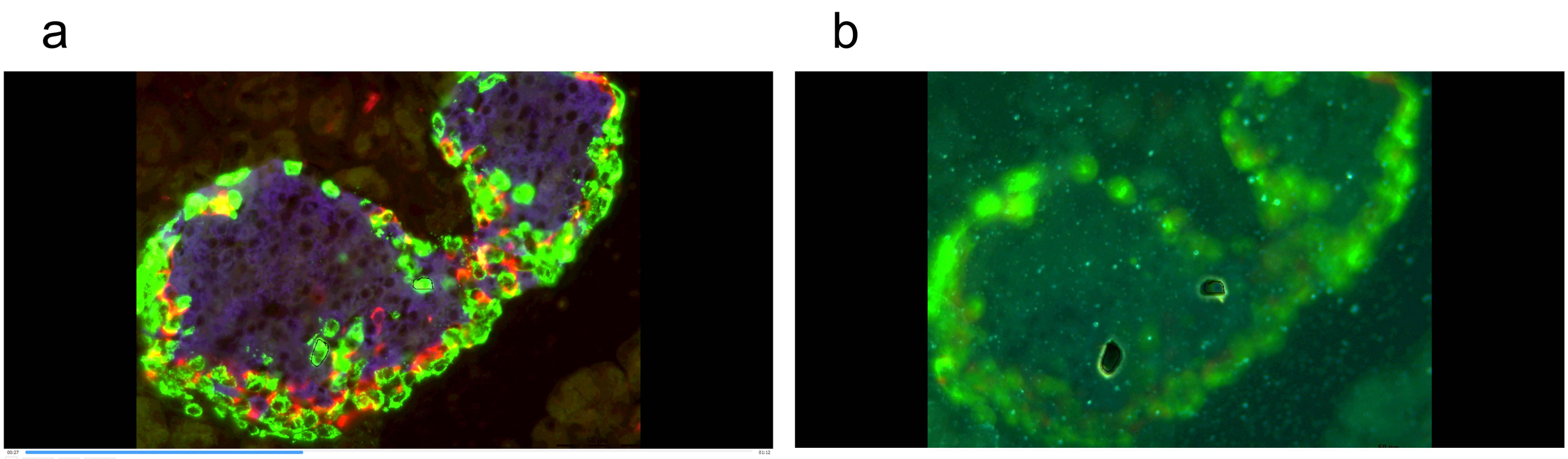

Supplementary Fig. 1

Supplementary Fig. 1. A clear resolution is achieved between the three endocrine cell types. a. Initial immunostaining of α-, β-, and δ-cells according to glucagon (green), insulin (blue), and somatostatin (red) staining, respectively. **b.** This clear staining allowed perfect cell separation, as demonstrated by the specific exclusion of individual α-cells.

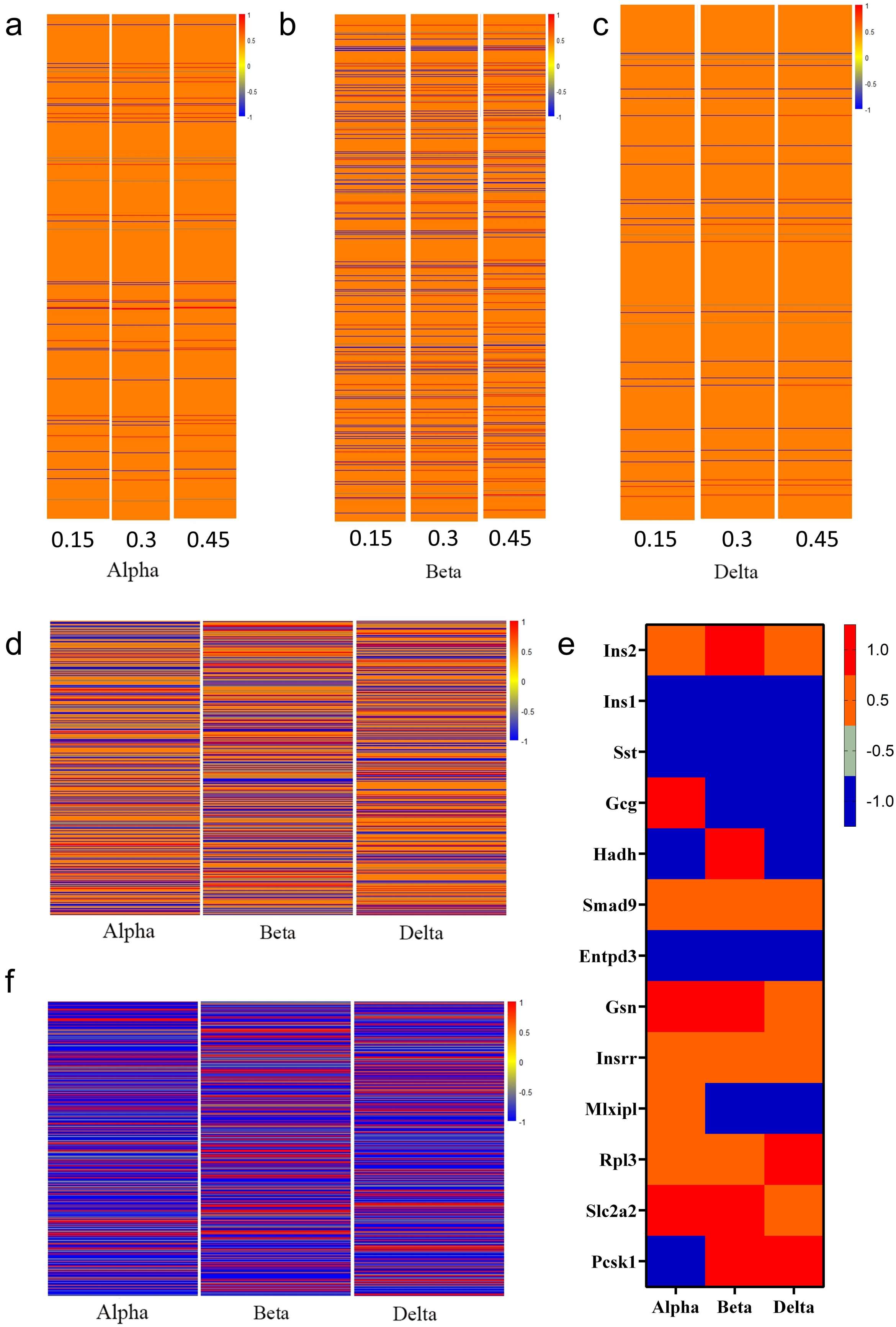

Supplementary Fig. 2

Supplementary Fig. 2. Comparison of our data, obtained by the mechanistic method to transcriptomic proteomic data obtained by proteolytic methods as described in the literature. a-c: Heatmap Comparisons of the transcriptomic data. As our data are larger than the transcriptomic data compiled by van Gurp *et al.*, comparisons that include genes that appear in our dataset and not in the compiled data are presented in orange for α-cells (a), β-cells (b), and δ-cells (c). These comparisons complement Fig 6a-c. **d-f:** Heatmap Comparisons of our proteomic data to the one generated by Thielert *et al.* Only proteins that displayed standard deviation ≤ 0.25 were analysed. **d.** As our data is larger than the Thielert *et al* data, many of the proteins are in orange (0.5), indicating proteins that appear in our data set but not in the Thielert *et al.* study. **e.** Heatmap Comparison of high profile genes, known to be specifically expressed in the different cell types, are presented. **f.** The same data as in (D), omitting the orange (0.5) proteins.

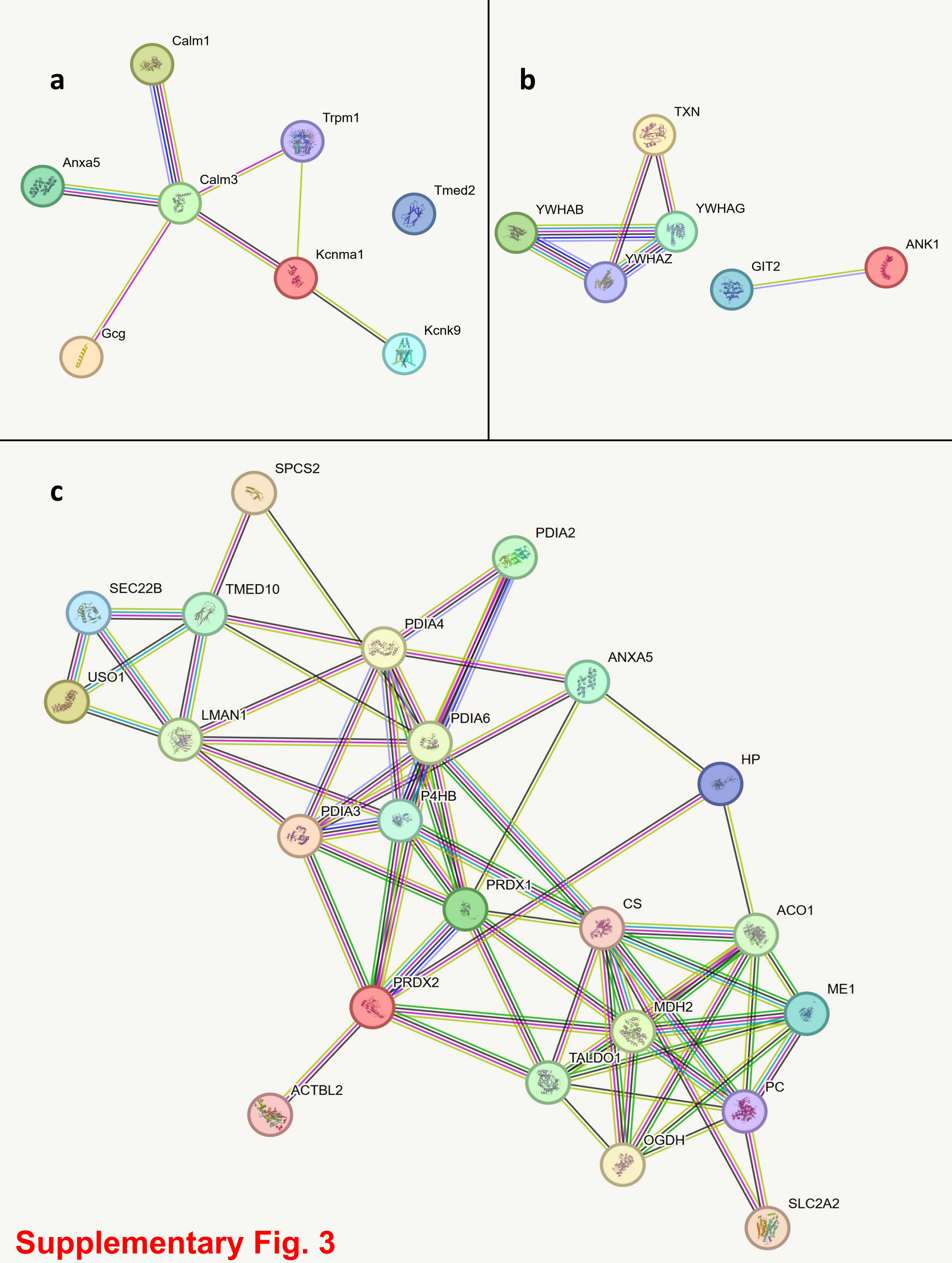

Supplementary Fig. 3

Supplementary Fig. 3. Detailed analysis of significant 'different' genes and proteins from our non-enzymatic dataset for α-, β-, and δ-cells. Significant genes and proteins identified as 'different' were analyzed using STRING with default settings[51]. Genes and proteins with known functions that are not commonly associated with multiple cell types (e.g., housekeeping genes, keratins, and histones) are highlighted for each cell type. The network connections are color-coded as follows: cyan lines represent curated database links, magenta lines indicate experimentally determined interactions, green lines denote gene neighborhood, red lines indicate gene fusions, blue lines represent gene co-occurrence, celadon lines are derived from text mining, black lines show co-expression, and purple lines indicate protein homology. **a.** Genes and proteins uniquely expressed in α-cells. **b.** Genes and proteins uniquely expressed in δ-cells. **c.** Genes and proteins uniquely expressed in β-cells.
